# Lymphotoxin Alpha (*LTA*) Polymorphism Is Associated with Prognosis of Non-Hodgkin’s Lymphoma in a Chinese Population

**DOI:** 10.1371/journal.pone.0066411

**Published:** 2013-06-20

**Authors:** Yan Zhang, Min-Bin Chen, Xiao-Yan Zhou, Xiao-Nan Hong

**Affiliations:** 1 Department of Medical Oncology, Kunshan First People’s Hospital Affiliated to Jiangsu University, Jiangsu, China; 2 Cancer Research Laboratory, Fudan University Shanghai Cancer Center, Shanghai, China; 3 Department of Medical Oncology, Fudan University Shanghai Cancer Center, Shanghai, China; Sapporo Medical University, Japan

## Abstract

**Background:**

Non-Hodgkin’s lymphoma (NHL) has been widely reported to be associated with autoimmune and pro-inflammatory response, and genetic polymorphisms of candidate genes involved in autoimmune and pro-inflammatory response may influence the survival and prognosis of NHL patients. To evaluate the role of such genetic variations in prognosis of NHL, we conducted this study in a Chinese population.

**Methods:**

We used the TaqMan assay to genotype six single nucleotide polymorphisms (SNPs) (*TNF* rs1799964T>C, *LTA* rs1800683G>A, *IL-10* rs1800872T>G, *LEP* rs2167270G>A, *LEPR* rs1327118C>G, *TNFAIP8* rs1045241C>T) for 215 NHL cases. Kaplan-Meier analysis was performed to compare progression free survival among two common genotypes. Cox proportional hazard models were used to identify independent risk factors.

**Results:**

We observed that *LTA* rs1800683G>A was significantly associated with risk of progression or relapse in NHL patients (HR = 1.63, 95%CI = 1.06–2.51; *P* = 0.028), particularly in Diffuse large B cell lymphoma (DLBCL) cases (HR = 1.50, 95%CI = 1.10–2.04, *P* = 0.01). Both univariate and multivariate Cox regression analysis showed that in DLBCL patients, Ann Arbor stage III/IV, elevated LDH level before treatment and *LTA* rs1800683 AA genotype carrier were independent risk factors for progression or relapse. While in NK/T cell lymphoma, Ann Arbor stage III/IV and elevated β_2_-MG level before treatment indicated poorer prognosis.

**Conclusions:**

The polymorphism of *LTA* rs1800683G>A contributes to NHL prognosis in a Chinese population. Further large-scale and well-designed studies are needed to confirm these results.

## Introduction

Non-Hodgkin’s lymphomas (NHLs) represent a heterogeneous group of malignancies that arise from the lymphoid system [1]. The most common subtype of NHL in China is diffuse large B cell lymphoma (DLBCL). In general, long-term remission can be achieved in approximately 50% of NHL patients with conventional chemotherapy [2]. But a number of patients do not achieve complete remission or relapse after chemotherapy. Several studies have shown that the courses of NHLs are variable, and NHL survival patterns vary by subtypes, suggesting different prognostic risk factors for NHL histological subtypes [3], [4], [5]. International prognostic index (IPI) model was developed for predicting outcome in patients with aggressive NHL in 1993, which pointed out that more than 60-year-old, advanced tumor stage, poor performance score, extranodal involvement and elevated lactate dehydrogenase (LDH) were adverse prognostic factors for NHL [6]. However, some patients with the same IPI score still show different survival and prognosis. The clinical characteristics involved in IPI do not reflect any biological information about NHL, either. We need more specific and inherited factors to predict NHL survival and prognosis personally.

Pro-inflammatory cytokines regulate the immune system by controlling lymphoid cell development and differentiation, and regulating the balance between the T-helper immune responses, as well as proliferation, differentiation, movement and communication between tumor and stromal cells [7]. Some studies support the hypothesis that common genetic variants in inflammation and immune-related genes can affect both the susceptibility and prognosis of NHL [8], [9], [10].

We have studied the association between inflammation and immune-related gene polymorphisms and risk of NHL previously [11]. We genotyped six SNPs (*TNF* rs1799964T>C, *LTA* rs1800683G>A, *IL–10* rs1800872T>G, *LEP* rs2167270G>A, *LEPR* rs1327118C>G, *TNFAIP8* rs1045241C>T) and found that one SNP (rs1045241) in *TNFAIP8* contributed to NHL susceptibility in a Chinese population. Subsequent stratification analyses by NHL subtypes showed that *TNFAIP8* rs1045241 T allele was significantly associated with an increased risk of DLBCL and follicular lymphoma (FL), but not NK/T-cell lymphoma. The results raised the possibility that inflammation and immune-related gene variants may have important roles for pathogenesis of specific NHL subtypes.

To further test the hypothesis that SNPs in inflammation and immune-related genes affect the prognosis of NHL patients, we evaluated the association between six SNPs mentioned and progression free survival (PFS) of NHL cases in a Chinese population. Table S1 had a detailed description of the six SNPs.

## Materials and Methods

### Ethics Statement

The study was approved by the Institutional Review Board of Fudan University Shanghai Cancer Center. Participation was voluntary. All participants singed a written informed consent, and all clinical investigation was conducted according to the principles expressed in the Declaration of Helsinki consent.

### Study Population

This study included 215 histologically confirmed NHL cases diagnosed between January 2008 and May 2011, 185 from Fudan University Shanghai Cancer Center and 30 from Kunshan First People’s Hospital Affiliated to Jiangsu University. The two centers are both in Eastern China and about 57 kilometers far apart. All patients were unrelated ethnic Han Chinese and had complete medical records. At recruitment, personal data from each participant regarding demographic information and clinical characteristics were collected via clinical record.

### Diagnosis and Staging

All cases were classified and reviewed according to the 2008 WHO classification of tumors of haematopoietic and lymphoid tissues [12]. The tumor staging was defined with Ann Arbor system.

### Genotyping

Genomic DNA was extracted from each blood sample by using the Qiagen Blood DNA Mini Kit (Qiagen Inc., Valencia, CA) according to the manufacturer’s instructions. DNA purity and concentration were determined by spectrophotometric measurement of absorbance at 260 and 280 nm by a UV spectrophotometer (Nano Drop Technologies, Inc., Wilmington, DE) and all DNA samples were suitable for genotyping.

All TaqMan assays for this study including the pre-designed SNP-genotyping assay mix containing PCR primers and probes were purchased from ABI (Applied Biosystems, Foster City, CA). Genotyping were conducted on the ABI 7900HT detection system (Applied Biosystems). To ensure the accuracy of genotyping results, four duplicated controls and four negative controls (no DNA) were included in each of the 384-well plates. The analyzed fiuorescence results were then auto-called into the genotypes using the built-in SDS2.2 software of the system.

### Statistical Analysis

The last follow-up date was February 28, 2012. PFS was calculated from the initiation treatment to the time of progression, relapse, death, or the last follow-up. Patients alive on the last follow-up date were considered censored. PFS was assessed using the Kaplan–Meier method and compared between risk groups using the log-rank test. Cox regression model was used in the univariate and multivariate analyses. Hazard ratios (HRs) and their 95% confidence intervals (CIs) were calculated. All statistical tests were two-sided, and *P*<0.05 was considered statistically significant. All analyses were performed using SPSS software, version 16.0 (SPSS Inc., Chicago, IL, USA).

## Results

### Patient Characteristics

The clinical characteristics of 215 NHL cases were summarized in Table 1. More males were enrolled, and the ratio was 1.69∶1. The median age was 48-year-old. In this study, the most common subtype of NHL was DLBCL (51.2%), followed by NK/T cell lymphoma (28.4%) and FL (11.1%).

**Table 1:** Clinical characteristics of 215 NHL cases.

Characteristics	Number	%
Sex		
Male	135	62.8
Female	80	37.2
Age at diagnosis		
Median (range)	48 (15–79)	
<60	163	75.8
≥60	52	24.2
Subtype		
DLBCL	110	51.2
NK/T-cell lymphoma	61	28.4
FL	24	11.1
Other B cell lymphomas	8	3.7
Other T cell lymphomas	12	5.6
Ann Arbor Stage		
I	60	27.9
II	74	34.4
III	46	21.4
IV	35	16.3
B symptom		
No	145	67.4
Yes	70	32.6
Elevated LDH level		
No	146	67.9
Yes	69	32.1
Elevated β_2_-MG level		
No	95	44.2
Yes	120	55.8
HBsAg positive		
No	171	79.5
Yes	44	20.5
Recruitment site		
Shanghai Cancer Center	185	86.0
Kunshan First People’s Hospital	30	14.0

During follow-up, 97 cases (45.1%) appeared progression, relapse or death. The median progression free survival was 16 months (0–60 months).

### Effect of SNP Genotypes on PFS by Subtypes

Frequencies of those six SNPs genotypes in NHL overall and subtypes are analyzed. One SNP in *LTA* (rs1800683) was significantly associated with risk of progression or relapse in NHL patients (HR = 1.63, 95%CI = 1.06–2.51; *P* = 0.028), but no associations were found between the other SNPs and PFS of NHL patients. Given that different subtypes may have different risk factors, we analyzed by subtypes. The quantities of some subtypes were so few that the statistical bias was inevitable, so we only analyzed PFS of DLBCL and NK/T cell lymphoma.

In DLBCL subtype, 110 cases were available for analysis. The median PFS was 15.5 months (0.5–40 months). Similar to the result of NHL overall, only the SNP *LTA* rs1800683G>A was slightly associated with risk of progression or relapse in DLBCL patients (*P* = 0.057 by log-rank test), but the other SNPs showed no association with PFS of DLBCL.

**Figure 1:**
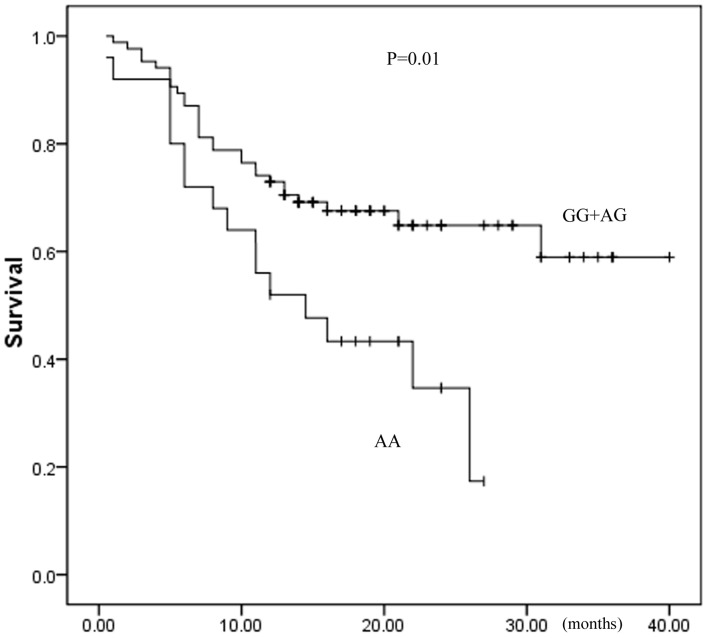
Association between LTA rs1800683G>A genotypes and PFS in DLBCL.

We further investigated the SNP *LTA* rs1800683G>A. When the *LTA* rs1800683 GG and AG genotype were grouped together and used as the reference group, *LTA* rs1800683 AA genotype was found to be associated with worse PFS of DLBCL patients, with the hazard ratios being 1.50 (95%CI: 1.10–2.04; *P* = 0.01), which was shown in Figure 1. The results indicated that carriers of *LTA* rs1800683 AA genotype among DLBCL patients have poorer prognosis, and may easier to progress or have a relapse.

There were 61 NK/T cell lymphoma cases enrolled for this part of study, with the median PFS of 16 months (0–60 months). However, no association was found between these six SNPs and risk of progression or relapse in NK/T cell lymphoma.

### Effect of SNP Genotypes on Response to Chemotherapy

The cases were treated with different regimens by different subtypes, and not every subtype can benefit from chemotherapy. So we only analyzed DLBCL subtype. A total of 110 DLBCL cases received CHOP (cyclophosphamide, doxorubicin/epirubicin, vincristine, and prednisone) or CHOP-like regimen as first-line treatment. Among them, 71 cases (64.5%) received rituximab plus CHOP (R-CHOP). After 6 cycles of treatment, therapeutic outcomes were estimated. Response rates were 50.9% for complete remission (CR) or unconfirmed CR (CRu), 34.6% for partial response (PR), 0.9% for stable disease (SD) and 13.6% for progression disease (PD). The overall response (CR+PR) rate was 85.5%.

The impact of SNPs on overall response rate was assessed in 110 DLBCL cases, but no statistical differences were found for all six SNPs (The data were shown in Table S2).

### Univariate and Multivariate Analysis for PFS

We also tested whether clinical characteristics contribute to progression free survival by subtypes. In DLBCL patients, Both univariate and multivariate Cox-regression model showed that Ann Arbor stage III/IV (Univariate: HR = 3.32, 95%CI = 1.81–6.10,*P*<0.001; Multivariate: HR = 5.04, 95%CI = 2.34–10.83,*P*<0.001), elevated LDH level before treatment (Univariate: HR = 3.19, 95%CI = 1.76–5.77,*P*<0.001; Multivariate: HR = 3.18, 95%CI = 1.48–6.83,*P* = 0.003) and *LTA* rs1800683 AA genotype carrier (Univariate: HR = 1.50, 95%CI = 1.10–2.04,*P* = 0.01; Multivariate: HR = 2.04, 95%CI = 1.01–4.14,*P* = 0.049) were independent risk factors for progression or relapse, while chemotherapy combined with rituximab may reduce the risk of progression or relapse (Univariate: HR = 0.43, 95%CI = 0.24–0.78, *P* = 0.005; Multivariate: HR = 0.40, 95%CI = 0.20–0.79,*P* = 0.008), as shown in Table 2.

**Table 2:** Univariate and multivariate analysis for PFS in DLBCL cases.

Variables	Univariate analysis		multivariate analysis	
	HR (95%CI)	*P*-value	HR (95%CI)	*P*-value
Sex (Male/Female)	0.65(0.36–1.16)	0.143	0.84(0.44–1.61)	0.591
Age (>60/≤60)	1.09(0.55–2.15)	0.813	1.18(0.55–2.58)	0.669
B symptom (Yes/No)	1.03(0.74–1.45)	0.853	0.80(0.35–1.81)	0.588
Stage (III/IV/I/II)	**3.32(1.81–6.10)**	**<0.001**	**5.04(2.34–10.83)**	**<0.001**
LDH (elevated/normal)	**3.19(1.76–5.77)**	**<0.001**	**3.18(1.48–6.83)**	**0.003**
β_2_-MG (elevated/normal)	1.61(0.88–2.94)	0.123	0.63(0.29–1.38)	0.249
HBsAg (+/−)	0.85(0.43–1.69)	0.649	0.47(0.22–1.01)	0.052
Immunophenotype (ABC/GCB)	1.22(0.60–2.47)	0.582	0.68(0.31–1.51)	0.344
Rituximab plus (Yes/No)	**0.43(0.24–0.78)**	**0.005**	**0.40(0.20–0.79)**	**0.008**
rs1799964 CC/TC+TT	1.07(0.53–2.17)	0.857	4.74(0.94–23.9)	0.059
rs1800683 AA/AG+GG	**1.50(1.10–2.04)**	**0.01**	**2.04(1.01–4.14)**	**0.049**
rs1800872 GG/TG+TT	0.68(0.41–1.13)	0.138	0.89(0.09–1.92)	0.076
rs2167270 AA/AG+GG	1.04(0.51–2.12)	0.904	0.84(0.18–3.84)	0.817
rs1327118 GG/GC+CC	4.58(0.07–9.14)	0.479	6.06(0.01–12.7)	0.967
rs1045241 TT/TC+CC	1.06(0.69–1.62)	0.806	1.85(0.70–4.88)	0.212

In NK/T cell lymphoma patients, we found that no characteristics except Ann Arbor stage III/IV (Univariate: HR = 5.50, 95%CI = 2.53–11.9, *P<*0.001; Multivariate: HR = 3.59, 95%CI = 1.45–8.87,*P* = 0.006) and elevated β_2_-MG level before treatment (Univariate: HR = 3.69, 95%CI = 1.60–8.54,*P* = 0.002; Multivariate: HR = 3.73, 95%CI = 1.37–10.17,*P* = 0.01) were the risk factors for poor prognosis when analyzed using univariate and multivariate Cox-regression model, as shown in Table 3.

**Table 3:** Univariate and multivariate analysis for PFS in NK/T-cell lymphoma cases.

Variables	Univariate analysis		multivariate analysis	
	HR (95%CI)	*P*-value	HR (95%CI)	*P*-value
Sex (Male/Female)	1.39(0.61–3.19)	0.439	1.42(0.55–3.65)	0.468
Age (>60/≤60)	1.63(0.67–3.96)	0.286	2.08(0.69–6.29)	0.196
B symptom (Yes/No)	0.67(0.34–1.30)	0.239	0.86(0.40–1.85)	0.697
Stage (III/IV/I/II)	**5.50(2.53–11.9)**	**<0.001**	**3.59(1.45–8.87)**	**0.006**
LDH (elevated/normal)	1.75(0.85–3.59)	0.127	0.93(0.37–2.33)	0.873
β_2_-MG (elevated/normal)	**3.69(1.60–8.54)**	**0.002**	**3.73(1.37–10.17)**	**0.01**
HBsAg (+/−)	0.81(0.31–2.10)	0.659	0.36(0.12–1.15)	0.084
rs1799964 CC/TC+TT	0.89(0.12–6.72)	0.910	8.16(0.01–13.7)	0.954
rs1800683 AA/AG+GG	1.72(0.82–3.61)	0.151	0.98(0.39–2.50)	0.973
rs1800872 GG/TG+TT	1.27(0.49–3.30)	0.619	1.57(0.47–5.25)	0.463
rs2167270 AA/AG+GG	0.29(0.04–2.09)	0.217	0.01(0.00–9.61)	0.960
rs1327118 GG/GC+CC	1.54(0.01–30.7)	0.425	1.10(0.01–11.2)	0.968
rs1045241 TT/TC+CC	2.07(0.49–8.63)	0.319	3.37(0.54–21.09)	0.193

## Discussion

The survival patterns of different histological subtypes of NHL are highly variable. Indolent lymphomas progress slowly and have long-term survival; while aggressive lymphomas may have poor prognosis that progress in a short time [1]. International prognostic index (IPI) is still known as the effective prognostic factor for aggressive lymphomas today [3], [6]. But more and more researchers have focused on the effect of inflammation and immune-related gene polymorphisms on pathogenesis and prognosis of NHL. The cytokines coded by these genes play a key role in immune-regulating. They control lymphoid cell development and differentiation, and regulate the balance between the T-helper immune responses, as well as proliferation, differentiation, and the movement and communication between tumor and stromal cells [13], [14], [15]. Given the biologic properties, we hypothesize that the variants in cytokine genes may alter transcription and expression, and further inhibit the cellular signaling pathways, as well as the balance of immunity. Finally, the survival and prognosis of NHL patients will be influenced by the changes of microenvironment.

Our results showed that *LTA* rs1800683 G>A was associated with progression free survival of DLBCL patients. The carriers of *LTA* rs1800683 AA genotype had poorer prognosis. This is the first study to report that this polymorphism can affect PFS of DLBCL in a Chinese population. Lymphotoxin-α (LT-α) is an important member of tumor necrosis factor (TNF) family. As a key mediator of inflammation through the induction of chemokines and vascular adhesion molecular, LT-α also play crucial roles in lymphoid organ development, as well as lymphangiogenesis [16]. Scientists have been interested in whether the variants in *LTA* gene contribute to lymphomagenesis and prognosis of NHL. Some researchers hold the idea that polymorphisms in *LTA* gene may increase the synthesis and release of LT-α, which lead to deregulation of NF-κB signaling. Constitutive NF-κB activation can promote continuous lymphocyte proliferation, survival and apoptosis [16], [17]. Several studies have reported another SNP *LTA* G252A (rs909253) was a prognostic predictor for NHL in white populations [18], [19]. Moreover, Chae believed that *LTA* C804A (rs1041981) was associated with poor treatment outcome in Korean DLBCL patients [20]. The result of our study also proved that *LTA* rs1800683G>A influences PFS in Chinese DLBCL patients, but the biological mechanism of this polymorphism needs further functional studies to verify.

Our result also showed that after 6 cycles of CHOP or R-CHOP chemotherapy in DLBCL patients, the CR rate was 50.9%, and the overall response rate was 85.5%, which was similar to the data reported by other studies [21], [22]. But we did not find any significant association between these six SNPs and the overall response rate. While, Warzocha et al. found the serum TNF-α level was higher in carriers of TNF/LTA haplotype than other patients, and they also have poorer therapeutic outcome [17]. Another study in France reported that compared with *IL-10* -1082AA genotype, patients with -1082 G allele had higher CR rate, longer five-year PFS and overall survival [23]. There are several potential explanations for the inconsistency. For one thing, ethnic and demographic differences between Chinese populations and other populations are exist, so frequencies of genotypes are different; for another, interactions among other genes and molecules may also play important roles in NHL prognosis, which means the association between a single SNP and prognosis may not be a linear relationship. The treatment outcome is affected by various factors, which should be taken into account, such as the living standard, performance status, side effects of chemotherapy and quality of caring. In our study, 62.3% cases are Ann Arbor stage I/II, and the short-term effect is better. This may explain why no difference was found for response rate. Moreover, 64.5% DLBCL patients were treated with CHOP plus rituximab, and the interaction between rituximab and polymorphisms should be considered.

The results of multivariate Cox-regression model analysis indicated that Ann Arbor stage III/IV, elevated LDH level before treatment and carriers of *LTA* rs1800683 AA genotype were independent prognostic risk factors for DLBCL patients, and chemotherapy combined with rituximab may reduce the risk of progression or relapse. For NK/T cell lymphoma patients, Ann Arbor stage III/IV and elevated β_2_-MG level before treatment were both the robust predictors of poor prognosis. It has been proved by other study that disease stage and LDH level are significantly associated with survival and prognosis of NHL patients [21]. Rituximab has also been reported to improve overall survival and progression-free survival on DLBCL patients. The result of MInT study showed after 6 cycles of CHOP or R-CHOP chemotherapy, R-CHOP group had improved time to treatment failure (TTF) than CHOP alone group (79% vs 59%), as well as overall survival [24]. Other large scale prospective studies, like GELA study and RICOVER-60 study also suggested that elder (>60 years old) DLBCL patients who received R-CHOP had longer event-free survival (EFS), PFS, disease-free survival (DFS) and OS than CHOP group [25], [26]. β_2_-microglobin (MG) is a non-specific tumor marker. Serum β_2_-MG level will elevate when the proliferation rate of lymphocyte is increased, which can be seen in multiple myeloma, malignant lymphoma and chronic lymphocytic leukemia. Previous study reported elevated serum β_2_-MG level could predict a relapse of DLBCL [27]. But few studies focused on the association between serum β_2_-MG level and prognosis of NK/T cell lymphoma. Our result suggested that serum β_2_-MG level was associated with the prognosis of NK/T cell lymphoma. Patients with elevated β_2_-MG before treatment had poorer prognosis and were easier to progress or relapse. But this conclusion should be verified with further clinical studies.

Despite of the strengths, our study also has limitations. First, we did not do the multiple testing corrections. If the p-value for *LTA* rs1800683 G>A was corrected by the Bonferroni method, it would no longer be significant. Second, we only discussed the effect of SNP on PFS and short-term outcome of NHL subtypes, but we did not analyze the overall survival. So our results might not comprehensively reflect the role of inflammation and immune-related gene polymorphisms. Third, the modest sized study is only powered to detect relatively large effects. We did not analyze other NHL subtypes except DLBCL and NK/T cell lymphoma due to extreme rarity. The genetic and clinical characteristics of different NHL subtypes can be quite different. Therefore, larger studies are warranted to confirm the effects of *LTA* rs1800683 SNP in other cohorts. In addition, we selected only one functional SNP for each candidate gene, which restricted further haplotype analysis. One SNP does not describe all the genetic variation in a gene, and optimal future studies should tag the variation in the gene and test haplotypes as well.

### Conclusions

In summary, our study showed for the first time that rs1800683G>A SNP in the 5′UTR of *LTA* gene was associated with PFS of DLBCL in a Chinese population. The carriers of rs1800683 AA genotype had poorer prognosis and progress/relapse more easily. These results support the hypothesis that SNPs in inflammation and immune-related genes can affect the prognosis of NHL patients. It will also provide a new insight into NHL and NHL subtypes prognosis and have potential implication in clinic care of NHL patients. However, replication of our findings in other studies is warranted to confirm its significance.

## Supporting Information

Table S1 **The information of six SNPs.**
(DOC)

Table S2 **The association between SNP genotypes and response rate to chemotherapy.**
(DOC)
