# Supplementary material for: Lymphotoxin Alpha (LTA) Polymorphism Is Associated with Prognosis of Non-Hodgkin’s Lymphoma in a Chinese Population
Source: PLoS One. 2013 Jun 20;8(6):e66411. doi: 10.1371/journal.pone.0066411 (PMC3688772; doi:10.1371/journal.pone.0066411)
Supplement: Table S1 — The information of six SNPs. (DOC) [file pone.0066411.s001.doc]

Table S1 The information of six SNPs

| **Gene** | **Nucleotide change** | **SNP**  **rs #** | **Chromosome** | **Putatively functional location in gene** | **Amino acid change** |
| --- | --- | --- | --- | --- | --- |
| TNF | T>C | rs1799964 | 6p21.33 | 5’near gene | No |
| LTA | G>A | rs1800683 | 6p21.33 | 5’UTR | No |
| IL-10 | T>G | rs1800872 | 1q31 | 5’near gene | No |
| LEP | A>G | rs2167270 | 7q31.3 | 5’UTR | No |
| LEPR | G>C | rs1327118 | 1p31 | 5’near gene | No |
| TNFAIP8 | C>T | rs1045241 | 5q23.1 | 3’UTR | No |
