# Supplementary material for: Lymphotoxin Alpha (LTA) Polymorphism Is Associated with Prognosis of Non-Hodgkin’s Lymphoma in a Chinese Population
Source: PLoS One. 2013 Jun 20;8(6):e66411. doi: 10.1371/journal.pone.0066411 (PMC3688772; doi:10.1371/journal.pone.0066411)
Supplement: Table S2 — The association between SNP genotypes and response rate to chemotherapy. (DOC) [file pone.0066411.s002.doc]

Table S2 The association between SNP genotypes and response rate to chemotherapy

| SNPs | case | | | CR/CRu + PR | | *P* value |
| --- | --- | --- | --- | --- | --- | --- |
|  | n | % | | n | % |  |
| *TNF* rs1799964T>C | | | |  |  | 0.723 |
| TT+CT | 105 | 95.5 | | 90 | 85.7 |  |
| CC | 5 | 4.5 | | 4 | 80 |  |
| *LTA* rs1800683G>A | | | |  |  | 0.379 |
| GG+AG | 85 | 77.3 | | 74 | 87.1 |  |
| AA | 25 | 22.7 | | 20 | 80 |  |
| *IL-10* rs1800872T>G | | | |  |  | 0.651 |
| TT+GT | 92 | 83.6 | | 78 | 84.8 |  |
| GG | 18 | 16.4 | | 16 | 88.9 |  |
| *LEP* rs2167270G>A | | | |  |  | 0.723 |
| GG+AG | 105 | | 95.5 | 90 | 85.7 |  |
| AA | 5 | | 4.5 | 4 | 80 |  |
| *LEPR* rs1327118C>G | | | |  |  | 0.06 |
| CC+CG | 108 | 98.2 | | 92 | 85.2 |  |
| GG | 2 | 1.8 | | 2 | 100 |  |
| *TNFAIP8* rs1045241C>T | | | |  |  | 0.802 |
| CC+CT | 94 | 85.5 | | 80 | 85.1 |  |
| TT | 16 | 14.5 | | 14 | 87.5 |  |
